# Are we using right dose of oxytocin?

**DOI:** 10.4103/0019-5049.71020

**Published:** 2010

**Authors:** D Devikarani, SS Harsoor

**Affiliations:** BMC & RI, Bangalore, India; 1 Editor, Indian Journal of Anaesthesia, #21, 2^nd^ Cross, Kirloskar Colony Basaveshwar Nagar, 2^nd^ stage, Bangalore, India. E-mail: harsoorss@hotmail.com

Postpartum haemorrhage (PPH) is one of the leading causes of maternal mortality. The incidence of PPH is estimated to be 10% of all deliveries.[1] The crucial aspect of the prevention and treatment of PPH is focussed around the appropriate use of uterotonic agents.

Among the various uterotonics, oxytocin (OT) is the most commonly used. It is routinely administered after delivery, whether spontaneous or operative, by bolus and infusion to initiate and maintain adequate uterine contractility after placental delivery, to minimise blood loss and prevent PPH. Prophylactic routine use of OT has been shown to reduce the incidence of PPH by up to 40%, implying that in every 22 women receiving OT, one PPH could be prevented.[2]

Several empirical regimens have been proposed for OT administration during caesarian delivery (CD), and this has led to many different practices in its administration worldwide. It is recommended to give 20 units of OT per litre of crystalloid infused at 10 ml/minute until the uterus contracts satisfactorily and bleeding is controlled, and then, the infusion is reduced to 1–2 ml/minute.[3] In a survey on the use of OT for CD, it was found that 295 out of 360 departments of anaesthesiology administer OT as a bolus (85.3%) and 48 (13.9%) give it as a slow infusion.[4] The dosage ranged from 1 to 80 IU and one out of eight departments administered 10 IU or more as bolus.

OT, a nonapeptide hormone and transmitter, is expressed in a variety of tissues, as are its receptors. Apart from uterus, oxytocin receptors (OTRs) are also found in heart and large vessels.[5] OT acts as a paracrine and/or autocrine mediator of various multiple biological effects *in vivo*. These effects are exerted primarily through interactions with G-protein coupled OT/vasopressin receptors which stimulate phospholipase C-mediated hydrolysis of phosphoinosides.[6] The OT used clinically is synthetic and identical to the hormone normally released from the posterior pituitary, but devoid of contamination by other polypeptide hormones and proteins found in natural proteins.[7]

In the uterus, the potent uterotonic actions of OT are mediated by the OTRs through G-protein activation to stimulate phospholipase C activity. The activated OTR increases the frequency of contraction and the force by sensitising the contractile apparatus of the myocytes to calcium.[8]

Because of the effects of oestrogen, the uterine OTR population density increases progressively during pregnancy to reach a peak at term. In late pregnancy (37–41 weeks), before the onset of labour, uterine OTR concentrations are on average 12 times higher than in early pregnancy and about 80 times higher than in the non-pregnant uterus. In cases of failed induction of labour with OT and in post-term pregnancies (43–46 weeks), the receptor concentration is significantly lower than that in spontaneous labour.[9] Around the onset of labour, uterine sensitivity to OT increases tremendously. OTR expressing smooth cells are observed diffusely and heterogeneously. The level of OTR transcripts increases according to the duration of pregnancy. The receptor messenger RNA rises 300-fold at parturition, compared with that of the non-pregnant myometrium.[10] Therefore, the uterus becomes very sensitive to the effects of OT and this could explain the uterine response to low doses of OT in non-labouring CD.[11] In early labour, the OTR population is significantly higher (2.5 times) than in term labouring patients. But when the cervix is more than 7 cm dilated, the OTR concentrations are reduced to just twice of those found in early pregnancy.[9]

Effective uterine contraction can be achieved after elective CD in non-labouring women at term by administering boluses of OT, no larger than 1 IU. The minimum effective IV bolus dose of OT is 0.35 IU,[11] while the necessary dose in labouring women at CD is about 9 times higher.[12] The difference is believed to be due to the reduction of OT-binding sites and desensitisation of myometrial OTRs in active labour. It has been found that ED90 of OT required to prevent uterine atony and PPH after an elective CD to be 0.29 IU/minute, or approximately 15 IU of OT in 1 l of intravenous fluids administered over 1 hour period. This OT infusion dose is 30% less than the clinical infusions currently in use.[13] Increasing the OT dose above 5 IU during elective CD has not been found to be advantageous.[14] It is observed that adequate uterine tone (UT) can be achieved with small bolus doses, 0.5–3 units of OT, but the incidence of hypotension increases significantly after 5 units. Hence, it is suggested that excessive doses of OT to achieve adequate UT during elective CD needs re-evaluation.[15]

Continuous exposure of human myometrial cells to OT leads to a significant loss in their capacity to respond to further doses of OT due to receptor desensitisation.[16] It is a usual practice to increase the dose of OT, assuming that higher doses will result in more effective uterine contraction. The higher doses of OT are unlikely to improve uterine contractions further and prevent PPH because the population of OTRs will not only be reduced but also be desensitised. The decrease in sensitivity is dependent upon the duration of OT exposure. This OT-induced desensitisation occurs over a clinically relevant time frame of 4.2 hours.[13] Hence, in labouring patients with haemorrhage, when response to OT is inadequate, consideration should be given to the alternate pathway of uterotonic medications such as ergot derivates, carboprost or misoprostol.

Studies in women undergoing CD for failed induction indicate that ED90 for OT is 2.99 IU. Hence, 3 IU of OT as a “loading dose” can achieve uterine contractions before a maintenance OT infusion (20 IU/l at 120 ml/hour) is continued.[12] It is recommended that this dose be administered as a rapid dilute infusion to avoid hypotension. Continued responsiveness of the cells to prostaglandin F2α stimulation 6 hours after OT pretreatment indicates that postreceptor signalling pathways were maintained.[16]

The usage of low-dose OT may reflect the method of assisted delivery of placenta, since it allows effective uterine contractions during foetus to placenta delivery interval.[12] Rapidly injected large doses of OT are known to produce various adverse effects such as hypotension, nausea, vomiting, chest pain, headache, flushing, myocardial ischaemia, ST-T segment changes, pulmonary oedema, and severe water intoxication with convulsions. The hypotensive action of OT demonstrated in animal models is believed to be mediated by the direct effect on OTRs in the cardia and larger vessels like aorta and vena cava by inducing release of Atrial Natriuretic Peptide (ANP).[5]

In a comparative study, there was a significant reduction in Mean Arterial Pressure (MAP), 30 seconds after administration of a 10 IU bolus OT, but a significant increase in heart rate (HR) and cardiac output (CO) occurred 1 minute after 5 U administration.[17] When 5 IU bolus IV was compared with 5 IU infusion, MAP decreased up to 27 mm of Hg and HR increased by 7 beats/minute at 35 seconds in the bolus group, which recovered to baseline at 110 seconds. The infusion group in contrast had a decrease in MAP of only 8 mm Hg and HR increased by 10 beats/minute.[18] Weis administered OT 0.1 U/kg intravenously to healthy pregnant women in the first trimester for elective termination of pregnancy and found that MAP and total peripheral resistance decreased approximately by 30% and 50%, respectively, but HR and Stroke Volume (SV) increased by 30% and 25%, respectively; thus, the CO increased to more than 50% above control.[19] Pulse power analysis conducted in a study demonstrated that hypotension in response to OT was associated with a decrease in systemic vascular resistance (SVR) and a compensatory increase in SV, HR, and CO.[20] Doses as small as 0.003 mg/kg were followed by hypotension. The reduction in MAP and speed of recovery were dose dependent.[17,21]

When OT 10 IU bolus IV was administered while 12 lead ECG, online vectorcardiography and invasive arterial blood pressure were monitored, it caused chest pain, transient profound tachycardia, hypotension and concomitant signs of myocardial ischaemia, according to marked ECG and STC-VM changes.[22] It was shown that slower injection of OT can effectively minimise the cardiovascular side effects without compromising the therapeutic benefits.[18] But these cardiovascular changes after bolus injection are usually mild (15–20%) and short lived and well tolerated by healthy women.[23] The liver, kidneys and the enzyme oxytocinase are responsible for the short half-life of OT.[1] A marked decrease in blood pressure may occur if OT is administered to a patient with blunted compensatory reflex responses, as may be produced by anaesthesia. Likewise, hypovolaemic patients may be particularly susceptible to OT induced hypotension. There is high incidence of intense flushing even after administering small doses of OT, illustrating the potent vasodilating properties of the drug.[11] It is seen that coadministration of phenylephrine obtunds OT-induced decreases in SVR and increases in HR and CO, but cannot abolish the unwanted haemodynamic effects of OT.[24]

OT exhibits a minimal anti diuretic hormone (ADH)-like activity.[7] ADH differs from OT in only two amino acids) which may be seen as water intoxication when administered in larger doses. The risk of this complication can be minimised by infusion of OT in an electrolyte containing solution like normal saline or Ringer’s Lactate. When OT is to be administered in high doses for a considerable length of time, the concentration should be increased rather than increasing the flow rate of a more diluted solution.[3]

OT remains the first line of treatment for prevention and treatment of PPH. Prophylactic OT administration and uterine massage are both important.[15] The use of 5 IU OT as a standard dose to achieve UT during elective CD is excessive and re-evaluation of dose requirement is necessary. Adequate uterine contraction can occur with lower doses of OT (0.5–3 units). Slower injection of OT can effectively minimise the cardiovascular side effects of a bolus dose without compromising the therapeutic benefits. Since OT has dose-dependent side effects, it appears prudent to administer OT slowly in infusions. In cases of haemorrhages, if there is no adequate response to initial treatment with OT, consideration must be given to the use of second line of uterotonics like ergot alkaloids, carboprost or misoprostol. Utmost restraint must be exercised while infusing in a haemodynamically unstable patient.
